# Impact of body mass index on survival outcome in patients with differentiated thyroid cancer^☆^

**DOI:** 10.1016/j.bjorl.2017.02.002

**Published:** 2017-02-28

**Authors:** Yousif Al-Ammar, Bader Al-Mansour, Omar Al-Rashood, Mutahir A. Tunio, Tahera Islam, Mushabbab Al-Asiri, Khalid Hussain Al-Qahtani

**Affiliations:** a King Saud University, College of Medicine, Department of Otolaryngology-Head & Neck Surgery, Riyadh, Saudi Arabia; b King Fahad Medical City, Radiation Oncology, Riyadh, Saudi Arabia; c King Saud University, College of Medicine and Research Center, Riyadh, Saudi Arabia; d King Fahad Medical City, Radiation Oncology Comprehensive Cancer Center, Riyadh, Saudi Arabia

**Keywords:** Differentiated thyroid cancers, Body mass index, Overall survival, Disease free survival, Câncer diferenciado de tireoide, Índice de massa corporal, Sobrevida global, Sobrevida livre de doença

## Abstract

**Introduction:**

Increased body mass index is known to be associated with the high prevalence of differentiated thyroid cancers; however data on its impact on survival outcome after thyroidectomy and adjuvant therapy is scanty.

**Objective:**

We aimed to evaluate the impact of body mass index on overall survival and disease free survival rates in patients with differentiated thyroid cancers.

**Methods:**

Between 2000 and 2011, 209 patients with differentiated thyroid cancers (papillary, follicular, hurthle cell) were treated with thyroidectomy followed by adjuvant radioactive iodine-131 therapy and thyroid-stimulating hormone suppression. Based on body mass index, patients were divided into five groups; (a) <18.5 kg/m^2^ (underweight); (b) 18.5–25 kg/m^2^ (normal weight); (c) 26–30 kg/m^2^ (overweight); (d) 31–40 kg/m^2^ (obese) and (e) >40 kg/m^2^ (morbid obese). Various demographic, clinical and treatment characteristics and related toxicity and outcomes (overall survival, and disease free survival) were analyzed and compared.

**Results:**

Median follow up period was 5.2 years (0.6–10). Mean body mass index was 31.3 kg/m^2^ (17–72); body mass index 31–40 kg/m^2^ was predominant (89 patients, 42.6%) followed by 26–30 kg/m^2^ seen in 58 patients (27.8%). A total of 18 locoregional recurrences (8.6%) and 12 distant metastasis (5.7%) were seen. The 10 year disease free survival and overall survival rates were 83.1% and 58.0% respectively. No significant impact of body mass index on overall survival or disease free survival rates was found (*p* = 0.081). Similarly, multivariate analysis showed that body mass index was not an independent prognostic factor for overall survival and disease free survival.

**Conclusion:**

Although body mass index can increase the risk of thyroid cancer, it has no impact on treatment outcome; however, further trials are warranted.

## Resumo

**Introdução:**

Sabe-se que o aumento do índice de massa corpórea está associado à alta prevalência de câncer diferenciado de tireoide; entretanto, os dados sobre seu impacto no desfecho de sobrevivência após tireoidectomia e terapia adjuvante são escassos.

**Objetivo:**

Objetivou-se avaliar o impacto do índice de massa corpórea nas taxas de sobrevida global e sobrevida livre de doença em pacientes com câncer diferenciado de tireoide.

**Método:**

Entre 2000 e 2011, 209 pacientes com câncer diferenciado de tireoide (papilar/folicular/de células de Hürthle) foram tratados através de tireoidectomia, seguida de tratamento com iodo radioativo-131 adjuvante e supressão de hormônio estimulante da tireoide. Com base no índice de massa corpórea, os pacientes foram divididos em cinco grupos; (a) <18,5 kg/m^2^ (baixo peso); (b) 18,5–25 kg/m^2^ (peso normal); (c) 26-30 kg/m^2^ (sobrepeso); (d) 31-40 kg/m^2^ (obesos) e (e) > 40 kg/m^2^ (obesos mórbidos). Várias características demográficas, clínicas e de tratamento e toxicidade associada e desfechos (sobrevida global e sobrevida livre de doença) foram analisadas e comparadas.

**Resultados:**

O período médio de acompanhamento foi de 5,2 anos (0,6-10). O índice de massa corpórea médio foi de 31,3 kg/m^2^ (17-72); o índice de massa corpórea de 31-40 kg/m^2^ foi predominante (89 pacientes, 42,6%), seguido por 26-30 kg/m^2^, observado em 58 pacientes (27,8%). Observaram-se 18 recidivas locorregionais (8,6%) e 12 metástases distantes (5,7%). As taxas de sobrevida livre de doença e sobrevida global de 10 anos foram de 83,1% e 58,0%, respectivamente. Não foi encontrado impacto significativo do índice de massa corpórea nas taxas de sobrevida global ou sobrevida livre de doença (*p* = 0,081). Da mesma forma, a análise multivariada mostrou que o índice de massa corpórea não foi um fator prognóstico independente para sobrevida global e sobrevida livre de doença.

**Conclusão:**

Embora o índice de massa corpórea possa aumentar o risco de câncer de tireoide, ele não tem impacto no resultado do tratamento; contudo, outros estudos são necessários.

## Introduction

The prevalence of overweight body mass index (BMI) >30 kg/m^2^ and obesity has increased worldwide during past decade and data has shown that 35% of the Americans are obese.^1^, ^2^ In Kingdom of Saudi Arabia, BMI is increasing in both sexes and across all ages with an overall prevalence of 44%.^3^, ^4^ Obesity is well known risk factor for various types of malignancies including endometrial carcinoma, colorectal carcinoma and breast carcinoma.^5^, ^6^ Recent data has reported the correlation of increased BMI with differentiated thyroid cancer (DTC).^7^ A recent review has shown that morbid obese patients (BMI > 35 kg/m^2^) were found to have significantly larger tumors than patients with BMI < 35 kg/m^2^.^8^ Similarly, another study from South Korea not only reported highest incidence of DTC in obese women but also correlation of DTC with higher mean waist circumference, fat ratio, and blood pressure.^9^

Although the causal relationship exists between BMI and DTC, its impact on treatment outcomes including disease free survival (DFS) and overall survival (OS) rates after thyroidectomy and Adjuvant radioactive iodine-131 (RAI) therapy and TSH suppression is not well known.

The purpose of present study was to evaluate the impact of BMI on locoregional control (LRC), distant metastasis control (DMC), DFS and OS, and toxicity profile in Saudi patients with DTC treated with thyroidectomy and adjuvant RAI therapy.

## Methods

After formal approval from the institutional ethical committee, medical records of 209 DTC patients, who were treated at our hospital during the period of July 2000 and December 2011, were reviewed using computer based database system.

### Demographic, clinicopathological and radiological data

Demographic and clinical data including age at the time of diagnosis, gender and symptomatology were reviewed. Different histopathological characteristics, including tumor size, histopathologic variants, multifocality, tumor, lymph node and metastasis (TNM) staging were recorded. Data was collected from different imaging modalities including neck Ultrasonography (USG), whole body I-131 scintigraphy (WBS), computed tomography (CT) scan of neck and chest and flourodeoxyglucose positron emission tomography (FDG-PET). Data regarding different treatment modalities including thyroidectomy, +/− neck dissection, adjuvant radioactive iodine-131 (RAI) ablation and its doses in millicurie (mCi) were also recorded.

### BMI calculation

For the purpose of study, each patient was categorized according to BMI. Height and weight were measured at the time of accrual using institutional protocols and BMI was calculated using the formula of weight in kilograms divided by the square of the height in meters (kg/m^2^). BMI was then categorized into five groups as follows: underweight as BMI < 18.5 kg/m^2^; normal weight as BMI from 18.5 to 25 kg/m^2^; overweight as BMI from 25 to 30 kg/m^2^; obese as BMI from 31 to 40 kg/m^2^ and morbid obese as BMI above 40 kg/m^2^.

### Statistical analysis

The primary endpoints were DFS and OS rates. Secondary points were; the comparative analysis of different clinicopathological features of DTC according to BMI categories, LRC and DMC rates. Local recurrence (LR) was defined as the duration between surgery date and date of clinically or radiologically detectable disease in the thyroid bed and/or in cervical lymph nodes on imaging (USG, WBS, CT and FDG-PET) after evaluation of elevated thyroglobulin (TG) levels. Distant Metastasis (DM) was defined as the duration between surgery date and date of documented disease outside the neck on imaging after evaluating for elevated TG. DFS was defined as the duration between surgery date and date of documented disease reappearance/relapse, death from cancer and/or last follow-up. OS was defined as the duration between surgery date and date of patient death or last follow-up.

Chi-square or Student's *t*-tests were used to determine the differences in various clinical variables. Probabilities of LRC, DMC, DFS and OS rates were shown with the Kaplan–Meier method and the comparison for various survival curves was performed using log rank. All statistical analyses were performed using the computer program SPSS version 16.0.

## Results

Patient's characteristics are shown in Table 1. There were a total of 209 patients, 165 (79.0%) females and 44 (21.0%) males. Classic type was the most predominant in 162 (77.5%) of patients. Multifocality was seen in 80 (38.3%), ETE was present in 41 (19.6%), and LVSI was present in 39 (18.6%) of patients. Surgical margins were positive in only 21 (10.1%) of patients. Lymph node metastasis was noted in 54 (25.8%) of patients. Background thyroid tissue was normal in 64 (30.6%), multinodular in 68 (32.6%), lymphocytic in 45 (21.5%) and Hashimoto's in 32 (15.3%). There were 189 (90.4%) who underwent near or total thyroidectomy, and the remaining 20 (9.6%) underwent lobectomy. Central neck dissection was done in 58 (27.7%) of patients. Near or total thyroidectomy was performed on most of the patients who had classic papillary thyroid carcinoma. Those who were referred from other hospital to this tertiary center after undergoing lobectomy, were treated by total thyroidectomy as completion. Lobectomy was reserved only for those individuals whose tumor was restricted only in one lobe and there was no evidence of intrathyroidal metastasis. Central neck dissection was performed in patients with enlarged lymph nodes at the time of surgery or identified on cervical ultrasonography.

**Table 1:** Patients’ characteristics.

Variable	Whole cohort – *n* (%)
*Total patients*	209
*Age (years)*	41.1 (16–78) SD ±11.6
≤45 years	127 (60.7)
≥45 years	82 (39.3)
*Gender*	
Female	165 (79.0)
Male	44 (21.0)
Female to male ratio	3.8
*Type of surgery*	
Near or total thyroidectomy	189 (90.4)
Lobectomy	20 (9.6)
*Lymph node surgery*	
Central neck dissection	58 (27.7)
Lateral neck dissection	28 (13.4)
Sampling	19 (9.1)
None	104 (49.7)
Mean size (cm)	2.3 (0.1–10.0) ± 12.4
*Histopathologic variants*	
Classic	162 (77.5)
Follicular	19 (9.1)
Hurthle cell	6 (2.8)
Tall cell	21 (10.1)
Sclerosing	1 (0.5)
*Multifocal*	
Yes	80 (38.3)
No	129 (61.7)
*ETE*	
Yes	41 (19.6)
No	168 (80.4)
*LVSI*	
Yes	39 (18.6)
No	170 (81.4)
*Surgical margins*	
Positive	21 (10.1)
Negative	188 (89.9)
*Lymph node metastasis*	
Yes	54 (25.8)
No	183 (87.6)
*Background thyroid tissue*	
Normal	64 (30.6)
Multi-nodular goiter	68 (32.6)
Lymphocytic thyroiditis	45 (21.5)
Hashimotos’ thyroiditis	32 (15.3)
Distant Metastasis at presentation	5 (2.4)
*AJCC staging*	
I	107 (51.2)
II	36 (17.2)
III	53 (25.4)
IV A	10 (4.8)
IV B	–
IVC	3 (1.4)
*Mean postoperative TG (ng/mL)*	1.39 (0.1–42,890)
*BMI (kg/m*^*2*^*) mean*	31.2 (17–72)
*BMI groups*	
<18.6	3 (1.4)
18.6–25	43 (20.6)
26–30	58 (27.8)
31–40	89 (42.6)
>40	16 (7.7)
*RAI dose*	
*No*	53 (25.4)
*30* *mCi*	64 (30.6)
*100* *mCi*	45 (21.5)
*150–200* *mCi*	47 (22.5)
*RT to neck*	12 (5.7)

*n*, number; SD, standard deviation; ETE, extrathyroid extension; LVSI, lymphovascular space invasion; AJCC, Americal Joint Commission on Cancer; TG, thyroglobulin; BMI, body mass index; RAI, radioactive iodine, mCi, millicurie; RT, radiation therapy.

### Complications and toxicities

Post-thyroidectomy complication rates were minimal; permanent hypocalcemia was seen in four patients (1.9%) and no correlation was seen with BMI (*p* = 0.063). Overall, RAI ablation was tolerated well without any Grade 3 or 4 side effects; however, acute and late (Grade – 3/4) complications were seen significantly 16 patients (7.66%) with no association with BMI (*p* = 0.71).

## Treatment outcomes

Median follow up period was 5.2 years (range: 0.6–10). For whole cohort, the 5 year LRC and DMC rates were 91.4% and 94.3% respectively. Total 18 LRs (8.6%) were observed; 2 in BMI 18.6–25 kg/m^2^, 9 in BMI 26–30 kg/m^2^, 4 in BMI 31–40 kg/m^2^ and 3 in BMI >40 kg/m^2^ (*p* = 0.051). The LRs were salvaged by surgery; lateral neck dissection (10 patients); completion thyroidectomy (4 patients) and excision (4 patients) followed by RAI ablation (14 patients). Similarly, total 12 DM (5.7%) were observed; 6 in BMI 26–30 kg/m^2^, 3 in BMI 31–40 kg/m^2^ and 3 in BMI >40 kg/m^2^ (*p* = 0.062). DMs were salvaged by RAI ablation and palliative irradiation (one patient).

**Figure 1:**
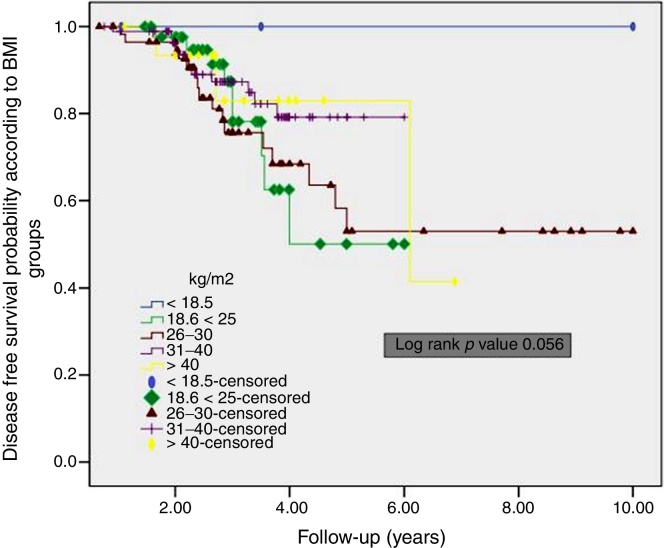
Kaplan–Meier curves of disease free survival according to BMI groups.

**Figure 2:**
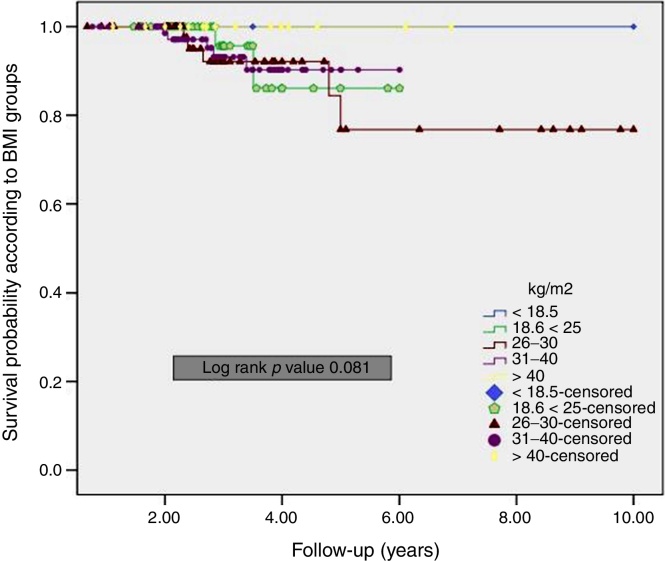
Kaplan–Meier curves of overall survival according to BMI groups.

The overall 5 year DFS rate was 92.8%. Furthermore, the DFS for the patients with BMI 18.6–25 kg/m^2^ was worse than those patients who have BMI of 26–30 kg/m^2^ and 31–40 kg/m^2^, although the difference was not significant (*p* = 0.056) (Fig. 1). The overall 5 year OS rate was 94.1%. No significant difference was in BMI groups (*p* = 0.081) (Fig. 2).

On multivariate analysis, important prognostic factors for DFS and OS were age, AJCC staging, lymph node involvement, LVSI and adjuvant RAI therapy. BMI was found not an important independent prognosticator (Table 2).

**Table 2:** Multivariate analysis of variables on disease free survival and overall survival.

Variable	Disease free survival		Overall survival	
	*p*-Value	OR (95% CI)	*p*-Value	OR (95% CI)
Age (<45 vs. 45 years)	0.033	0.83 (0.90–2.50)	0.041	0.50 (0.10–2.41)
Cormorbids (yes vs. no)	0.091	1.28 (1.07–1.97)	1.00	1.80 (0.79–2.10)
AJCC stage (<II vs. >II)	0.041	0.67 (0.60–1.34)	0.01	0.85 (0.80–1.90)
N stage (N0 vs. N1)	0.033	0.81 (0.79–2.00)	0.051	1.21 (1.10–2.10)
BMI kg/m^2^ (>30 vs. <30)	0.052	1.15 (1.0–2.45)	0.061	1.15 (1.01–1.65)
LVI (no vs. yes)	0.031	0.91 (0.76–1.45)	0.60	1.10 (0.89–2.00)
Adjuvant RAI (yes vs. no)	0.031	0.50 (0.10–2.41)	0.041	0.50 (0.67–2.81)

OR, odds ratio; 95% CI, 95% confidence intervals; AJCC, American Joint Commission on Cancer; N, node; BMI, body mass index; LVI, lymphovascular invasion; RAI, radioactive iodine.

## Discussion

Obesity is one of the endemic Health issues in the developed countries. It is a known risk factor for several diseases, an increased risk of developing endometrial, prostate, breast, pancreatic and thyroid cancer exists in the obese.^10^ In our study we tried to sort out the correlation between BMI and survival rates in patients with DTC. To the best of our knowledge, this is the first study to mention impact of BMI on the prognosis of patients with DTC.

Our findings suggest that BMI does not have an impact on OS of DTC patients treated surgically. Although our results did show that the DFS in the underweight patient's was worse than the normal and overweight group, it did not reach statistical significance. Further, univariate and multivariate analyses revealed age and AJCC staging as independent prognostic factor for both DFS and OS. Also N stage of the disease, lymphovascular invasion and adjuvant radioactive iodine was significantly associated with DFS in univariate and multivariate analysis (Table 2).

Impact of BMI on survival outcomes has been studied in various malignancies such as breast and gastric cancers.^11^, ^12^, ^13^ However, few studies have evaluated the BMI and obesity as a prognostic factor in head and neck malignancies.^14^ Takenaka et al. identified pretreatment BMI as an independent prognostic factor for survival among patients with head and neck squamous cell carcinoma (HNSCC) treated with chemoradiation, in their study the population was divided in to three groups: underweight (18.5 kg/m^2^ < BMI),normal weight (18.5–25 kg/m^2^) and overweight (25 kg/m^2^ > BMI), and they noted that the overweight patients had the most favorable prognosis, and the underweight patients the worst.^15^ Similarly, studies by Shen et al.,^16^ and Huang et al.,^17^ found high BMI to be strongly associated with better overall survival, disease-specific survival and failure-free survival. Data of both the studies suggested BMI as an independent prognostic factor in patients with nasopharyngeal carcinoma (NPC). However Lin et al.^18^ study did not reveal any association between pretreatment BMI and overall survival, disease specific survival, distant metastasis free survival, or locoregional free survival in patients with NPC (Table 3)

**Table 3:** Summary of effect of BMI on head and neck cancer patients.

Authors, Years	BMI categories	Treatment modality	Effect of BMI
Takenaka et al.,^15^ 2015	Obese or overweight (25 kg/m^2^), normal (18.5 kg/m^2^ and <25 kg/m^2^), and underweight (<18.5 kg/m^2^).	Surgery, CRT, RT	192 surgically treated patients no statistically significant the effect of BMI on overall survival.
			In other treatment modalities high BMI was associated with a better prognosis.
Huang PY et al.,^17^ 2013	Obese (27.5 kg/m^2^), overweight (23.0–27.4 kg/m^2^), normal weight (18.5–22.9 kg/m^2^), underweight (<18.5 kg/m^2^).	IC + CCRT	Higher BMI was associated with increased failure free survival and overall survival.
		IC + RT	No influence on the risk of locoregional recurrences.
Lin YH et al.,^18^ 2015	Two groups (<23 kg/m^2^ vs. ≥23 kg/m^2^)	IMRT, CCRT, RT/CCRT + IC	BMI was not significantly associated with overall survival, disease specific survival, distant metastasis free survival, or locoregional free survival.
van Bokhorst–de van der Schuer B. et al.,^21^ 1999	BMI not calculated, Percentage of weight loss during the 6 months before treatment, the percentage of ideal body weight, serum albumin, total lymphocyte count, nutritional index, and bioelectrical impedance analysis.	Surgery	None of the studied nutritional parameters were associated with survival.
Present study	Morbid obese (>40 kg/m^2^), obese (31–40 kg/m^2^), overweight (26–30 kg/m^2^), normal weight (18.5–25 kg/m^2^), underweight (<18.5 kg/m^2^).	Surgery	BMI was not significantly associated with overall survival, disease free survival

CRT, chemoradiation therapy; RT, radiation therapy; CCRT, concurrent CRT; BMI, body mass index; IC, induction chemotherapy; IMRT, intensity-modulated radiotherapy.

Several studies suggested similar to our findings that other risk factors, such as age and stage;^15^ nodal status, aggressive histopathologic variants, multifocality, ETE, LVSI and adjuvant RAI are more important clinicopathological predictors than BMI in DTC.^19^, ^20^

Takenaka et al.^15^ inferred that different treatment modalities influences the impact of BMI on prognosis. This might explain the disparity between the results of different studies, including our study. Small number of studies discussed the BMI as a prognostic factor in head and neck malignancies treated by surgery. A study by Takenaka et al.^15^ and another by van Bokhorst-de van der Schuer et al.,^21^ involved 192 and 64 patients respectively, with head and neck malignances treated surgically, concluded that the impact of BMI on the prognosis was not statistically significant. Whereas the OS was significantly better in the patients with higher pretreatment BMI receiving chemoradiation and radiation therapy. Moreover BMI did not come up as an independent prognostic factor in the result of Cox proportional hazard analysis in surgically treated patients.^15^

Several reasons were explained in the studies showing a positive impact of BMI on the survival of patients with head and neck malignancies. First of all, the modality of treatment, patients with head and neck malignancies treated by chemotherapy and/or radiation were found to have a more favorable prognosis if they were in a high BMI group, however, this relation was not seen in patients treated surgically.^15^, ^17^ Secondly, Cancer Cachexia which is a known challenge in treating patients with malignancies such as pancreatic, gastric and head and neck cancers.^17^, ^22^ Cachexia can decrease treatment response and affect the immune system capability in fighting infections, which leads to death.^17^, ^22^ In our study the possible reasons for the insignificant findings were: the treatment which was by Surgery and (RAI) ablation, with a 94.1% 5 year OS rate and cancer cachexia which was not seen in the study group. Additionally In our cohort, post thyroidectomy complications were minimal and patients tolerated adjuvant RAI ablation very well with minimal toxicity.

Our study had several limitations, first the retrospective nature of present study and second the small sample size of 209 patients. Besides these two, our study population is mainly Middle Eastern or Asian descendants, additional studies in other populations are warranted.

## Conclusion

In conclusion, although BMI is known to increase the risk of thyroid cancer, and it is a strong prognostic factor in the head and neck cancers associated with cachexia, and treated with chemoradiation or radiation therapy; it is not a strong predictor for the treatment outcome in DTC patients.

## Conflicts of interest

The authors declare no conflicts of interest.
